# 造血干细胞移植后EB病毒相关淋巴增殖性疾病中国专家共识（2022年版）

**DOI:** 10.3760/cma.j.issn.0253-2727.2022.09.002

**Published:** 2022-09

**Authors:** 中华医学会血液学分会 中国医师协会血液科医师分会

造血干细胞移植（hematopoietic stem cell transplantation, HSCT）是许多血液系统疾病最重要的根治手段[1]–[5]。随着HSCT技术的进步及临床广泛的推广应用，许多血液病患者可获得长期无病生存[6]–[11]。感染相关性疾病是HSCT后的常见并发症[12]，EB病毒（EBV）相关移植后淋巴增殖性疾病（post-transplant lymphoproliferative disorders, PTLD）是一种严重的并发症。中华医学会血液学分会与中国医师协会血液科医师分会联合组织国内相关领域专家制定本共识，旨在建立并优化EBV-PTLD的临床诊治路径，为我国HSCT的临床实践提供规范、优化的诊治指导建议。

一、EBV-PTLD概况

EBV-PTLD是指移植后患者由于免疫抑制而导致EBV感染或再激活后发生的一组表现为良性淋巴组织增殖或恶性肿瘤的异质性淋巴系统增殖性疾病[13]–[17]。

EBV-PTLD是HSCT后发生的最严重并发症之一，其发病与患者免疫功能抑制和EBV感染相关。正常情况下，EBV感染后可潜伏于淋巴细胞中，主要受控于EBV特异性的T细胞，使感染的细胞不致过度增殖[18]。HSCT后的患者处于免疫抑制状态，EBV特异性T细胞缺失或功能受损，在免疫监控降低的情况下EBV诱发淋巴细胞增殖从而导致PTLD[15],[18]。EBV主要感染B细胞，也有部分EBV感染T细胞或NK细胞而形成T/NK细胞亚型的病变。HSCT后EBV-PTLD大部分来源于供者，供者血清阳性时发生EBV-PTLD的风险更高[14],[19]。除EBV外，其他病毒也可能参与PTLD的发病。

二、EBV-PTLD的流行病学特征

不同移植类型、不同移植中心的EBV-PTLD发生率不尽相同。国内外研究数据显示，allo-HSCT后EBV-PTLD发生率为0.8％～11.9％[20]–[23]，中位发生时间为移植后55～63 d[24]–[26]，全相合供者移植、单倍体移植、非血缘供者移植后EBV-PTLD的发生率分别为0.3％～2.2％、3.4％～23.5％、2.3％～2.7％[21]–[23]。HSCT后1～5个月为PTLD的发病高峰期（每年每万例患者中发生120例），发病最高峰在HSCT后2～3个月（每年每万例患者中发生210例）[27]。自体造血干细胞移植后EBV-PTLD仅有个案报道[28]–[31]。

三、EBV-PTLD的危险因素

移植前危险因素包括：①体内或体外T细胞去除[13],[27],[32]–[34]；②患者年龄≥50岁[14]；③供、患者HLA配型不合[14],[35]；④供、患者EBV IgG状态不合（尤其供+/受−）[16],[35]；⑤强化预处理[36]；⑥含放疗的预处理[37]；⑦脾切除术后[35]；⑧二次移植[14]；⑨原发病为重型再生障碍性贫血等[13]–[14],[26],[32],[35]–[43]。

移植后危险因素包括：①Ⅲ/Ⅳ度急性移植物抗宿主病（GVHD）[35]；②中、重度慢性GVHD[13]；③CMV-DNA血症[16],[24]；④移植后30 d CD8^+^T细胞重建不良[24]。

四、EBV-PTLD的临床表现和诊断

（一）临床表现

发热和局灶或全身淋巴结肿大是EBV-PTLD最常见的临床表现，中枢神经系统、胃肠道等结外器官受累也十分常见，临床表现可不典型[44]–[45]。

（二）EBV相关监测

对于EBV-PTLD高危的患者，需要规律监测血EBV-DNA。常用实时荧光定量PCR方法监测外周血EBV-DNA载量[13],[46]–[47]。血清病毒含量低于血浆，血浆标本在反映EBV复制程度、PTLD活动性和治疗反应方面更为准确[48]–[50]，因此通常采用血浆或全血标本用于EBV-DNA检测[48],[50]–[54]，也有移植中心报道监测外周血单个核细胞内EBV-DNA载量[55]–[58]。既往有EBV感染的患者，病毒可持续潜伏在细胞内，因此单个核细胞中的EBV定量不能准确反映病毒再激活状态。allo-HSCT后1个月内或中性粒细胞植入后可开始监测EBV-DNA，每周至少1次，直到移植后3～4个月[13]–[14],[46],[50]。若外周血EBV-DNA载量升高，需增加监测频率[13]–[14],[46]。对于单倍体造血干细胞移植以及移植后出现重度GVHD的患者，需要延长外周血EBV-DNA的监测时间至半年以上[13],[46]。各移植中心可根据患者的具体情况决定监测频率和随访时间，进行个体化监测。

需要注意的是，部分患者可出现外周血和组织EBV分离现象，即外周血EBV-DNA检测阴性，但受累组织器官或分泌物、组织液标本检测为阳性。受累组织器官（如支气管肺泡灌洗液或脑脊液）检查证实EBV存在，可作为诊断的辅助依据。

建议：移植后1个月内或中性粒细胞植入后即开始采用定量PCR监测外周血EBV-DNA载量，监测频率及随访时间根据患者的病情确定。

（三）影像学检查

淋巴结彩超可以简单快速地评估浅表淋巴结肿大并初步判断其性质，增强CT有助于发现深部淋巴结肿大及结外病灶，这两者均为EBV-PTLD患者重要影像学评估手段。由于EBV-PTLD多为^18^F-FDG亲和的淋巴瘤，因此PET-CT在发现PTLD的结外病灶、临床分期及疗效评估方面更有优势[59]–[63]，其诊断PTLD的灵敏度、特异度分别为89％、89％，阳性预测值、阴性预测值分别为91％、87％[64]。

推荐：可采用彩超、CT和PET-CT作为EBV-PTLD的影像学诊断方法。

（四）组织病理学分型

EBV-PTLD的病理分型参考WHO 2016淋巴肿瘤分类，可分为：

（1）非结构破坏性PTLD：浆细胞增生；传染性单核细胞增多症；滤泡过度增生。

（2）多形性PTLD。

（3）单形性PTLD：①B细胞肿瘤：弥漫大B细胞淋巴瘤、伯基特淋巴瘤、浆细胞骨髓瘤、浆细胞瘤等；②T细胞肿瘤：外周T细胞淋巴瘤-非特指型、肝脾T细胞淋巴瘤等。

（4）经典型霍奇金淋巴瘤PTLD。

（五）临床分期

临床上可将EBV-PTLD简单分为结内和结外病变以及局限（单一病灶）和进展（多病灶）期疾病[13]。EBV-PTLD的分期也可参照Ann Arbor标准。对于^18^F-FDG亲和性淋巴瘤患者，推荐依据Lugano标准对EBV-PTLD进行分期[13],[65]–[66]。

（六）EBV-PTLD的诊断标准

受累淋巴结或结外器官活检病理是EBV-PTLD诊断的金标准，对于没有条件或不能耐受活检的患者，PET-CT有助诊断[13]。

明确EBV-PTLD的诊断需要进行以下检查项目：①临床症状和体征：包括是否有发热、淋巴结及其他器官肿大；②PET-CT/增强CT检查或彩超；③如有胃肠道症状，需要完善胃肠内镜检查；④组织活检病理学检查，包括EB病毒编码的RNA-原位杂交（EBER-ISH）、病毒抗原免疫组化和流式细胞学检测；⑤外周血EBV-DNA定量PCR检测[13]。

参考欧洲白血病抗感染学会（ECIL6）[13]和欧洲骨髓移植学会（EBMT）标准并结合国内外临床实践[24]–[25],[67]，可将EBV-PTLD分为“确诊的EBV-PTLD（proven EBV-PTLD）”和“临床诊断的EBV-PTLD（probable EBV-PTLD）”。

符合下述临床表现和实验室检查[13],[46]可诊断为“确诊的EBV-PTLD”：

（1）临床表现：发热、淋巴结肿大、肝脾肿大或其他结外器官受累的表现；

（2）实验室检查：组织活检病理需满足以下①、②中的任意1条和③：①原有组织结构被增生的淋巴组织破坏；②采用细胞和（或）病毒标志物发现单克隆或寡克隆细胞群；③采用检测DNA、RNA或蛋白的方法在组织样本中发现EBV感染的证据（仅有血EBV-DNA阳性不能诊断EBV-PTLD）[68]。

由于EBV-PTLD临床表现多样，早期可缺乏典型临床表现，但可迅速进展。具有定量监测拷贝数升高的EBV-DNA血症伴明显的淋巴结肿大、肝脾肿大或其他结外器官受累表现，排除其他原因后，可考虑为“临床诊断的EBV-PTLD”[13],[46],[67]。

EBV-PTLD诊断时还须注意与恶性血液病髓外复发、继发肿瘤及其他感染性疾病引起的淋巴组织增生相鉴别。

推荐：具有相关临床表现且有组织活检病理结果，可诊断为“确诊的EBV-PTLD”；具有相关临床表现和EBV-DNA血症但不能获得组织活检病理结果且排除其他原因，可诊断为“临床诊断的EBV-PTLD”。

（七）危险分层

根据移植类型和是否含有危险因素，将患者分低危、标危和高危，详见表1。

**表1:** EB病毒相关淋巴增殖性疾病（EBV-PTLD）危险分层

高危
非血缘供者造血干细胞移植
替代供者造血干细胞移植
同胞全相合造血干细胞移植（含至少1个危险因素）
标危
同胞全相合造血干细胞移植（无危险因素）
单倍体造血干细胞移植（后置环磷酰胺预处理方案）
低危
自体造血干细胞移植

对于有一个或多个PTLD危险因素的患者，需要加强外周血EBV-DNA的监测（表2），并适当延长移植后PTLD的随访时间。

**表2:** EB病毒相关淋巴增殖性疾病（EBV-PTLD）监测及诊断相关推荐

EBV-PTLD监测及诊断相关推荐：
①移植后1个月内或中性粒细胞植入后即开始采用定量PCR监测外周血EBV-DNA拷贝数，监测频率及随访时间根据患者的病情而定
②对于有1个或多个PTLD危险因素的患者，需要加强外周血EBV-DNA的监测至半年以上
③EBV-PTLD诊断时可采用彩超、CT和/或PET-CT检查进行影像学评估
④确诊的EBV-PTLD：存在PTLD临床表现且有组织活检病理结果支持的患者
⑤临床诊断的EBV-PTLD：同时存在PTLD临床表现及EBV-DNA血症，但无组织活检病理证据并除外其他原因的患者

五、EBV-PTLD的预防

（一）定义及适用人群

EBV-PTLD预防是指对于移植前EBV血清学阳性且具有EBV-PTLD高危因素的患者，移植后在血EBV-DNA阴性且没有临床症状时即给予预防性干预。

（二）利妥昔单抗

与对照组相比，allo-HSCT后预防性使用利妥昔单抗仅能降低EBV-DNA血症的发生率，不能降低EBV-PTLD的发生率[56]。目前国内外均缺乏临床证据表明利妥昔单抗可以预防allo-HSCT后的EBV-PTLD。

（三）细胞治疗

对于移植后高危患者，有条件的单位可以给予预防性EBV特异性细胞毒T细胞（EBV-CTL）输注[13]–[14]。研究表明，接受第三方EBV-CTL输注可以预防EBV-PTLD[69]。但国内尚无临床证据表明EBV-CTL输注可以有效预防EBV-PTLD。

（四）抗病毒药物

常用针对疱疹病毒的药物（阿昔洛韦、更昔洛韦等）对体内潜伏的EBV无效[70]。目前无临床证据表明常用抗疱疹病毒药物可以预防EBV-PTLD[13],[46]–[47]。

建议：目前尚无公认有效的预防策略，预防性治疗的有效性尚有争议。

六、基于EBV监测的抢先治疗

（一）抢先治疗的定义及适用人群

抢先治疗是指对于移植后EBV-PTLD的高危患者出现EBV-DNA血症但尚无临床表现时即给予治疗[13],[15]–[16]。抢先治疗的目标是及时清除血循环中的EBV，预防EBV-PTLD的发生。

（二）启动抢先治疗的EBV-DNA阈值

对于启动抢先治疗的外周血EBV-DNA载量，目前尚无公认的推荐阈值[13],[47]。出现血EBV-DNA阳性（高于生物参考值上限）的患者需要增加监测频率，出现不同日连续2次阳性时可启动抢先治疗。对于EBV-DNA拷贝数快速上升的患者，接受抢先治疗的临床意义可能更大[13],[71]。

推荐：目前无公认的启动抢先治疗的外周血EBV-DNA阈值。对于EBV-DNA拷贝数快速上升的患者，启动抢先治疗的临床意义更大。

（三）抢先治疗

1.免疫抑制剂减量：免疫抑制剂减量是EBV-PTLD患者重要的抢先治疗[13],[67]，有利于外周血循环EBV-DNA的清除[72]。

2. 利妥昔单抗：利妥昔单抗可有效降低EBV-PTLD的发生率，尤其在血EBV-DNA水平较高（如≥10^4^拷贝/ml）的患者中。治疗后EBV-DNA转阴率达到83％～100％，PTLD的发生率降至0～25％[41],[55],[58],[73]–[79]。利妥昔单抗与免疫抑制剂减量联用可以提高疗效。

利妥昔单抗的用法：375 mg/m^2^每周1次，直到EBV-DNA血症转阴[13],[26],[41],[55],[58],[73]–[76],[78],[80]–[81]。通常1～3次可转阴[55],[58],[73]–[74],[80]，一般使用1～4次[26],[36],[55],[75]–[76],[78],[81]，额外的剂量可能导致CD20表达下调而降低疗效。

有报道显示较低剂量利妥昔单抗（100 mg/m^2^）抢先治疗的疗效不低于标准剂量[82]。

3. EBV-CTL输注：EBV-CTL输注在国际上是重要的EBV-PTLD治疗手段，国内目前尚处于临床试验阶段。供者或第三方EBV-CTL均可安全、有效地清除外周血中的EBV-DNA[13]。研究表明，第三方EBV-CTL抢先治疗后，EBV-DNA转阴率为92％且无EBV-PTLD发生[69]。

推荐：利妥昔单抗是最重要的抢先治疗手段，建议联合免疫抑制剂减量。有条件的移植中心也可采用EBV-CTL输注进行抢先治疗（表3）。

**表3:** EB病毒相关淋巴增殖性疾病（EBV-PTLD）预防及抢先治疗推荐

EBV-PTLD预防及抢先治疗推荐：
①预防是指对于移植前EBV血清学阳性且具有EBV-PTLD高危因素的患者，移植后在血EBV-DNA阴性、尚无临床表现时即给予预防
②目前尚无公认的有效预防策略且预防性治疗的有效性尚有争议
③目前对于启动抢先治疗的外周血EBV-DNA载量无公认的推荐阈值，各移植中心可定义高于生物参考值上限为EBV-DNA阳性
④出现不同日连续2次外周血EBV-DNA阳性时可启动抢先治疗，对于EBV-DNA拷贝数快速上升的患者，启动抢先治疗的临床意义更大
⑤利妥昔单抗是最重要的抢先治疗手段，建议联合免疫抑制剂减量。有条件的中心也可采用EBV特异性细胞毒T细胞（EBV-CTL）输注进行抢先治疗

七、EBV-PTLD的治疗

（一）一线治疗

1.免疫抑制剂减量：单独采用免疫抑制剂减量治疗EBV-PTLD有效率较低，尤其对病理诊断为淋巴瘤的患者效果更差，通常和其他治疗方式联合使用以平衡GVHD风险[67],[83]–[84]。减量或停用时需警惕发生严重GVHD。

2. 利妥昔单抗：利妥昔单抗是EBV-PTLD的一线治疗选择[13],[15]–[16],[63]。研究表明，在免疫抑制剂减量的基础上使用利妥昔单抗，缓解率可达84％，明显高于免疫抑制剂未减量的患者（缓解率61％）[67]。有条件的移植中心可进行PTLD细胞来源检测，非B细胞来源或不表达CD20的PTLD患者不推荐使用利妥昔单抗。

利妥昔单抗的推荐用法：375 mg/m^2^每周1次[13]–[14],[63],[71],[85]–[88]。由于多次使用利妥昔单抗会使B细胞表面CD20表达下调而影响疗效[57]，利妥昔单抗通常使用1～4次[13],[63]。

影响利妥昔单抗疗效的因素：治疗后血EBV-DNA拷贝数无下降甚至进一步升高是治疗失败的最重要危险因素[25],[67]。此外，影响疗效的危险因素还包括年龄大于30岁、Ⅱ度以上急性GVHD、存在结外病灶、初始外周血EBV-DNA≥10^4^拷贝/ml以及无免疫抑制剂减量[67]。

（二）二线治疗

1. 细胞治疗

（1）EBV-CTL：EBV-CTL是EBV-PTLD的重要治疗手段，供者来源EBV-CTL的完全缓解率可达50.0％～84.6％[69],[89]–[93]。国内目前尚处于临床试验阶段。

参加临床试验的EBV-PTLD患者，对于接受脐血移植、不能获得干细胞二次捐献以及EBV血清学阴性的供者，可考虑采用第三方EBV-CTL[94]–[97]。EBV-PTLD患者接受第三方EBV-CTL输注后5周、6个月的总反应率分别为64％、52％，完全缓解率分别为36％、42％[95]；随访4～9年后，总生存率为59％[96]。利妥昔单抗治疗无效的EBV-PTLD患者，接受第三方EBV-CTL输注后，总反应率为68％，完全缓解率为57％[98]。

建议：若参加临床试验，每次可输注EBV-CTL（1～2）×10^6^/kg，每1～2周输注1次，可输注3～8次[93],[95],[98]–[99]。

临床研究的初步结果显示，发热在输注EBV-CTL 5～14 d后缓解，血EBV-DNA可在输注3～10 d后显著下降，终末器官损害的临床表现在输注8～15 d后显著改善，影像学异常在输注后约3周得到改善[93]。EBV-CTL输注的不良反应较少，GVHD发生较罕见[93],[95],[98]。

（2）供者淋巴细胞输注（DLI）：也可选择DLI代替临床试验的EBV-CTL[100]。研究表明，EBV-PTLD患者接受DLI后完全缓解率为56.7％，总反应率为73％ [93]。

DLI的用法：每次回输单个核细胞数（0.5～1.0）×10^8^/kg[101]，CD3^+^T细胞（0.2～2.0）×10^6^/kg[93],[99]，可输注1次或多次。

中位起效时间为2（1～5）d，淋巴结缩小的中位时间为6（1～14）d。DLI有导致重度GVHD的风险，Ⅱ～Ⅳ度急性GVHD发生率为14％～57.1％[93],[100]，DLI后2～4周内应保持免疫抑制剂在有效的预防浓度[100]。

推荐：利妥昔单抗是EBV-PTLD的一线治疗，需要联合免疫抑制剂减量；也可采用DLI治疗或参加EBV-CTL的临床试验。

2. 化疗±利妥昔单抗：化疗治疗EBV-PTLD的有效率为26.7％～74.0％[83],[101]，但化疗相关不良反应的发生率超过50％，化疗相关死亡率达26％[101]。一线治疗无效的患者，可采用常用的淋巴瘤化疗方案[15],[63],[102]。

3. 手术切除和局部放疗：孤立病灶的EBV-PTLD药物治疗效果不佳时，可考虑手术切除和（或）局部放疗[13],[63]。

推荐：对于一线治疗效果不佳的EBV-PTLD患者，可考虑化疗、手术切除及局部放疗等治疗方法。

（三）中枢神经系统EBV-PTLD的治疗

中枢神经系统EBV-PTLD的发生率约为3％[103]。治疗方法包括：①利妥昔单抗联合高剂量甲氨蝶呤和（或）阿糖胞苷为基础的化疗（须根据患者体能状态及合并症评估其耐受性和临床风险）[13],[104]–[106]。②利妥昔单抗全身用药或鞘内注射。有经验的中心，鞘内注射可从10～20 mg开始，每周1次，剂量可逐次递增，成人单次最高剂量不超过50 mg[13],[107]–[108]。③EBV-CTL输注[13],[109]。④局部病灶放疗[13],[110]。

EB病毒相关EBV-PTLD治疗推荐见表4。

**表4:** EB病毒相关淋巴增殖性疾病（EBV-PTLD）治疗推荐

EBV-PTLD治疗推荐：
①一线治疗中，单独免疫抑制剂减量效果不佳，通常和其他治疗方式联合使用以平衡GVHD风险，减量或停用时需警惕发生严重GVHD
②利妥昔单抗是EBV-PTLD的一线治疗，需要同时减量免疫抑制剂；也可采用DLI治疗或参加EBV-CTL的临床试验
③有条件的移植中心可进行PTLD细胞来源检测；非B细胞来源或不表达CD20的PTLD不推荐使用利妥昔单抗治疗
④对于一线治疗效果不佳的EBV-PTLD患者，可考虑化疗、手术切除及局部放疗等治疗方法

八、疗效评估

主要采用Lugano2014淋巴瘤疗效评估标准，对于FDG亲和性的PTLD采用PET-CT（Deauville标准）评价代谢缓解[111]–[112]；对于FDG非亲和性的PTLD采用CT和（或）磁共振检查评估影像学反应。

治疗反应包括：

完全缓解：①CT评估所有靶病灶完全消失，淋巴结靶病灶长径≤1.5 cm；②PET-CT扫描结果，Deauville评分1～3分；③无新发病灶。

部分缓解：①CT评估最多6枚淋巴结和结外靶病灶垂直直径乘积之和降低≥50％；②PET-CT扫描结果，Deauville评分4～5分，但代谢值低于基线；③无新发病灶。

疾病稳定：①CT评估最多6枚淋巴结和结外靶病灶长径与对应垂直直径乘积之和降低<50％；②PET-CT扫描，Deauville评分4～5分，但代谢较基线值相比无明显变化；③无新发病灶。

疾病进展：①CT评估1枚淋巴结和（或）结外靶病灶需符合以下异常条件：淋巴结和（或）结外病灶长径>1.5 cm，且长径与对应垂直直径乘积之和较最小状态增加≥50％；②PET-CT扫描Deauville评分4～5分，但代谢值高于基线；③出现新发病灶。

九、预后

EBV-PTLD是allo-HSCT后少见但严重的并发症，可爆发性进展，如不能得到及时诊治，死亡率可高达60％～80％[21],[113]–[114]。但给予及时、合理的治疗，患者远期生存率可接近60％[67]，因此重视EBV-PTLD防治对于保障移植安全和改善患者预后具有重大意义。
